# Effect of porcine plasma on growth and health of Holstein calves

**DOI:** 10.3168/jdsc.2021-0112

**Published:** 2021-10-09

**Authors:** D.R. Wood, R.M. Blome, L.C. Ribeiro, A.J. Keunen, B.W. Keunen, J.D. Crenshaw, J.M. Campbell, D.L. Renaud

**Affiliations:** 1 Animix, Juneau, WI 53039; 2 Mapleview Agri, Palmerston, ON, Canada N0G 2P0; 3 APC Inc., Ankeny, IA 50021; 4 Department of Population Medicine, University of Guelph, Guelph, ON, Canada N1S 2W1

## Abstract

The objective of this trial was to compare the health and performance of calves provided high feeding rates of a whey-based all-milk-protein calf milk replacer (MR) with those fed an MR containing either 5% or 10% porcine plasma, which replaced, respectively, either 15% or 30% of the whey-based proteins in the MR formula. A total of 320 male Holstein calves weighing a mean (± SD) of 47.8 ± 4.1 kg were sourced from local dairy farms, auction facilities, and local order buyers. Calves arrived at the research facility in 4 batches of 80 animals each and were randomly assigned to 1 of 3 groups: (1) MR composed of whey-based milk proteins (control group); (2) whey replacement of 5% spray-dried porcine plasma (replacing 15% of protein); or (3) whey replacement of 10% spray-dried porcine plasma (replacing 30% of protein). Calves were housed in individual pens for the first 56 d of the experiment and offered, twice daily, a 26% crude protein (CP), 20% fat MR standardized using synthetic amino acids to 2.4% lysine, 0.8% methionine, and 1.6% threonine. Amounts of MR offered from wk 1 to 8 were 0.65, 0.78, 0.91, 1.04, 1.04, 0.78, 0.52, and 0.325 kg/d, respectively. Calves were also offered a 20% CP texturized calf starter from d 0 to 56 and then transitioned over 7 d (d 56–62) of a 50% calf starter and 50% corn and pellet ration with 2% straw to a corn and pellet ration with 2% straw (18.1% CP) for the remainder of the experiment (d 63–77). Calves were individually weighed upon arrival, weekly through d 56, and at d 77. Grain was fed ad libitum, and remaining grain was weighed weekly to determine weekly consumption. Remaining grain was discarded and was replaced with fresh grain that was weighed and recorded. All milk was offered individually via bucket twice daily, and refusals were recorded following milk feeding by weighing back the remaining unconsumed milk solution. Calves were health scored twice daily, and any medical treatments or mortality were recorded. Time to mortality and medical treatments were analyzed using survival analysis, health scoring data were analyzed using a generalized linear model, and growth was evaluated using a mixed repeated-measures linear regression model. No differences in mortality or incidence of diarrhea were noted between groups. Although there was a high incidence of respiratory disease (65%), no differences were found between groups. Over the entire experimental period, calves gained 67.0 ± 14.9 kg; however, no differences in growth among groups were noted, with the exception that, on d 77, BW was greater for calves in the control group (115.8 ± 15.5 kg) compared with those fed a MR with 5% porcine plasma (113.4 ± 17.8 kg). No differences were found between groups with respect to feed conversion. In this study, an MR composed of either 5% or 10% spray-dried porcine plasma performed comparably to a whey protein–based MR.

## Highlights

• Calves were fed 40.6 kg of milk replacer (MR; 26% protein and 20% fat) powder over 8 wk.
• MR contained all-milk protein (control) or 15% or 30% protein from spray-dried porcine plasma (SDPP).
• Inclusion rates of SDPP were 0, 5, or 10% of the respective absolute MR formula (i.e., 0, 50, or 100 kg per 1,000 kg of MR powder).
• No differences were noted in morbidity or mortality between the groups.
• No differences in feed conversion or BW were noted, except at 77 d where control calves had a greater average BW than calves fed 5% SDPP.

## Graphical Abstract

**Figure:**
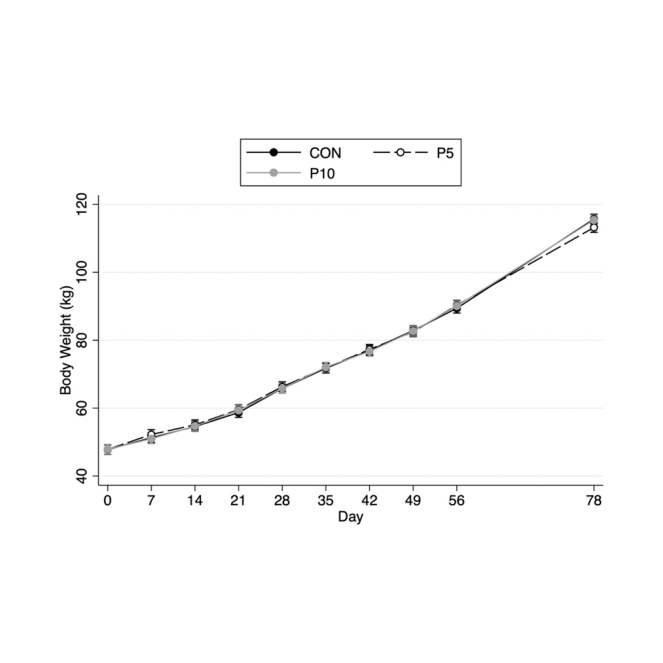
**Summary:** Spray-dried bovine plasma is shown to perform comparably to whey-based proteins when replacing CP in formula. However, research on using porcine plasma in milk replacers is limited. The objective of this study was to evaluate increased inclusion rates of porcine plasma in milk replacers. We demonstrated that Holstein calves fed high rates of milk replacer and replacing either 15% (P5) or 30% (P10) of the whey-based CP with spray-dried porcine plasma had comparable feed conversion, BW gain, mortality, and incidence of respiratory and enteric disease compared with calves fed an all-milk-protein formula (CON).

Spray-dried plasma protein has long been used in calf milk replacer (**MR**) formulas, and studies have shown that it performs comparably to milk proteins when milk replacers are fed at increased feeding rates (Morrison et al., 2017; Grice et al., 2020), even up to a 10% inclusion rate of plasma protein (Ziegler et al., 2018). However, most of the studies have evaluated spray-dried bovine plasma, with few validating the use of spray-dried porcine plasma (**SDPP**). In studies using SDPP, it has been shown to reduce calf mortality and days with diarrhea when used at 5% inclusion in the formula, replacing 20% of the CP compared with a whey protein concentrate-based formula (Quigley and Wolfe, 2003). In addition, when replacing 15% of whey-based proteins, SDPP was shown to reduce the severity of diarrhea and provide comparable performance (Wood et al., 2019).

The objective of this randomized controlled trial was to compare the health and performance of calves provided high feeding rates of a whey-based all-milk-protein MR with that of calves fed an MR composed of either 5% or 10% SDPP replacing, respectively, either 15% or 30% of the whey-based proteins in the MR formula. We hypothesized that the addition of SDPP would result in comparable performance to a whey-based formula even when replacing 30% of the whey-based protein. We also hypothesized that the addition of SDPP would reduce the incidence and severity of diarrhea and that increased inclusion rates of SDPP would further reduce diarrhea.

This randomized clinical trial was conducted between February and June 2020 at Mapleview Agri Ltd., a grain-fed veal facility used for commercial milk replacer and additive research located in southwestern Ontario, Canada. This facility was selected because of the availability of research technicians and ability to monitor the outcomes of the trial. Male Holstein calves, estimated to be between 5 and 14 d of age, arrived in 4 successive batches of 80 animals 21 d apart and were placed in a separate, mechanically ventilated, climate-controlled room within the facility where they stayed for the duration of the study. Calves were sourced directly from local dairy farms, order buyers, and auction facilities in Ontario. Calves were individually housed in 1-m^2^ individual stalls for the milk feeding period; however, after weaning on d 57, the calves were commingled within the same group of 5 consecutive calves that were individually housed preweaning (as described below). All calves were reared on the same production site for this study. The study was conducted in accordance with the *Guide for the Care and Use of Agricultural Animals in Research and Teaching* (FASS, 2010).

Upon arrival, calves were randomly assigned by researchers to a stall number between 1 and 80 according to a randomization command in Excel (Microsoft Corp.). Groups of 5 consecutive individually housed calves were randomly allotted to 1 of 3 MR treatments: (1) all milk protein (**CON**), (2) 15% of CP replaced with SDPP (Nutrapro P, APC Inc.) included at 5% of the absolute formula (**P5**), or (3) 30% of CP replaced with SDPP included at 10% of the absolute formula (**P10**). Within each room, there were 16 groups of 5 consecutive individually housed calves (hereafter referred to as a “pod”). Pods of each treatment group were evenly dispersed within the respective 80-calf room.

Milk replacers (Mapleview Milk Replacer, Mapleview Agri Ltd.) were blended to include the ingredients for each treatment group before the start of the study. Care was taken to ensure Lys, Met, and Thr were balanced equally in all 3 diets by using the analyzed AA content of the protein-containing ingredients and synthetic l-lysine, dl-methionine, and l-threonine to meet formula requirements. All MR were nonmedicated, void of any additives, and formulated to contain 26% CP, 20% fat, 2.4% Lys, 0.8% Met, and 1.6% Thr on an as-fed basis. All calves were bucket-fed at daily feeding rates of 650 g in 5 L of solution wk 1, 780 g in 6 L for wk 2, 910 g in 7 L for wk 3, 1,040 g in 8 L for wk 4 and wk 5, 780 g in 6 L wk 6, 520 g in 4 L wk 7, and 325 g in 2.5 L wk 8 (reported on an as-fed basis) with feedings split evenly between a.m. and p.m. Milk refusals were recorded twice daily following milk feeding and measured on an individual calf-level basis. No group medications or antimicrobials were administered.

Calves were provided free-choice water for the entire experiment and offered texturized calf starter (20% CP, as-fed basis) upon arrival until wk 8, and then transitioned to corn and pellet ration with 2% straw (18.1% CP, as-fed basis) for the remainder of the trial (Wallenstein Feed and Supply Ltd.). Grain concentrate was fed ad libitum and refusal weights were recorded at the end of each week. Grain was fed in a trough where the calves in each individual pod would have access; as such, grain intake was measured based on the intake of the pod.

Body weight was recorded at arrival and at 7, 14, 21, 28, 35, 42, 49, 56, and 77 d after arrival, using a digital scale (Tru-Test). Feed efficiency was calculated for the entire experimental period (0 to 77 d after arrival), the preweaning period (0 to 56 d after arrival), and the postweaning period (56 to 77 d after arrival), using the total amount of BW gained in each pod divided by the feed consumed (MR and concentrate) on an as-fed basis within each pod. Feed consumption was calculated for each pod on an as-fed basis because specific DM values were not available for all feeds consumed in the experiment.

Calves were scored daily for fecal consistency in the first 28 d of the experimental period as described by Renaud et al. (2020), where 0 = normal; 1 = soft, piles but spreads slightly; 2 = runny, spreads easily; and 3 = watery, liquid consistency, splatters. Calves with a fecal score of 2 or 3 were classified as positive for diarrhea and treated once daily at noon with electrolyte therapy (Truvitalyte, TruVital Animal Health) until recovered. Calves with respiratory disease were diagnosed and treated using a scoring system developed by University of California–Davis (Love et al., 2014). If the score was greater than 5, the calf was medically treated. Antimicrobial treatment and supportive therapy (nonsteroidal anti-inflammatory medications and intravenous fluids) was also recorded for each calf. The field technicians, calf-care staff, and statistician responsible for data analysis were blinded to the identity of the treatment groups.

All statistical analyses were conducted in Stata 16 (StataCorp). Descriptive statistics were generated on all explanatory variables in the data set. A one-way ANOVA was used to evaluate differences in BW and serum total protein at arrival as well as ADG between treatment groups. As the amount of MR consumed and refused as well as grain intake were not normally distributed, a Kruskal-Wallis test was used to identify statistical differences between treatment groups. A χ^2^ test was used to determine if there were differences between the source of calves or the level of failed transfer of passive immunity (**FTPI**), which was defined as a serum total protein of <5.1 g/dL (Renaud et al., 2018). Several explanatory multivariable models were created to explore the variables contained within the data set. For continuous variables, the assumption of linearity was evaluated graphically in each model; if a variable failed to meet the linearity assumption, the variable was categorized into quartiles. Repeated-measure linear regression models were created to evaluate the effect that treatment group had on growth following enrollment, whereas mixed linear regression models were used to evaluate feed efficiency. To evaluate the percent of days at risk with a fecal score ≥2 or a fecal score of 3, a generalized linear model with a logit link and binomial family was used. Cox proportional hazard models were created to evaluate the effect that treatment group had on mortality, treatment for diarrhea, and treatment for respiratory disease in the experimental period. In all models, the room the calves were housed in was included as a random effect or forced into the model to account for differences that could have occurred between the rooms within the experimental facility. In addition, in models evaluating BW, the pod of calves was included as an additional random effect. For the mixed linear model, homoscedasticity, normality of the BLUPs, and residuals were evaluated for model fit. The assumption of proportionality was assessed for the Cox proportional hazard models by using the test of proportional assumptions. Variables were considered significant at *P* ≤ 0.05 and a tendency at *P* > 0.05 to < 0.10.

A total of 320 calves were enrolled in the trial, with 110, 105, and 105 calves assigned randomly to the CON, P5 and P10 groups, respectively. Because of an odd number of groups compared with treatment size and the constraint of pods of 5 calves assigned to each treatment, 25 calves were enrolled for 2 of the treatments and 30 calves were enrolled for the third treatment within each room. The mean (± SD) BW of the calves at arrival was 47.6 ± 4.1 kg and it did not differ between treatment groups (*P* = 0.24). A total of 129 calves were sourced from local dairy farms, 132 calves were sourced from auction, and 59 were sourced from an order buyer direct from local farms. No differences were found among treatment groups with respect to source (*P* = 0.74).

Blood samples were taken upon enrollment from 240 of the 320 calves in the study, and a digital refractometer was used to determine serum total protein. Unfortunately, blood samples were not taken on calves in 1 of the 4 rooms because of regional COVID-19 restrictions. The mean level of serum total protein at arrival was 5.73 ± 0.67 g/dL, and no differences were found between the groups (*P* = 0.12). A total of 45 calves had FTPI, reflecting 21.3, 16.3, and 18.8% of calves in the CON, P5, and P10 groups, respectively. No differences in incidence of FTPI were found between groups (*P* = 0.72).

Eleven calves (3.4%) died during the trial: 2.7% in CON, 2.9% in P5, and 4.8% in P10. No differences were found between treatment groups using a log-rank test of survivor function (*P* = 0.66). Four calves died from respiratory disease, 4 died from diarrhea, 1 died from dehydration, 1 was euthanized because of lameness, and 1 died suddenly because of a neurological condition. No differences were found between P5 [hazard ratio (**HR**): 1.05; 95% CI: 0.21–5.22; *P* = 0.95] or P10 (HR: 1.76; 95% CI: 0.42–7.37; *P* = 0.43) and the CON group with respect to mortality, and no associations were found between BW at arrival or source of calves and mortality.

The number of days with a fecal score ≥2 was 1.6, 2.1, and 2.2 in the CON, P5, and P10 groups, respectively, which was not different between groups (*P* = 0.12). Calves spent 6.1, 7.6, and 8.4% of the first 28 d with a fecal score ≥2 in the CON, P5, and P10 groups, respectively. There was a tendency for the P10 group to have a higher proportion of days with a fecal score ≥2 (Table 1). Body weight at arrival and source of calves were associated with the proportion of days with a fecal score ≥2 (Table 1). The number of days with a fecal score of 3 was 0.51, 0.61, and 0.57 in the CON, P5, and P10 groups, respectively. No differences were identified between treatment groups (*P* = 0.77). Calves had a fecal score of 3 for 2.0, 2.2, and 2.5% of the first 28 d in the CON, P5, and P10 groups, respectively. No differences were found with respect to treatment group; however, arrival weight was associated with the proportion of days with a fecal score of 3, where calves weighing >50.8 kg had a lower proportion of days with a fecal score of 3 (relative proportion ratio: 0.33; 95% CI: 0.19–0.59; *P* < 0.001) compared with calves weighing <44.5 kg at arrival.

**Table 1:** Generalized linear regression model evaluating proportion of time with a fecal score ≥2 in the first 28 d following arrival in 320 calves randomly assigned to 1 of 3 milk replacers^1^

Variable	Proportion ratio	Coefficient	*P*-value	95% CI
Treatment group				
CON	Referent			
P5	1.29	0.26	0.14	−0.08 to 0.59
P10	1.39	0.33	0.07	−0.03 to 0.68
BW at arrival				
<44.5 kg	Referent			
44.5 to 47.6 kg	0.79	−0.23	0.26	−0.62 to 0.17
47.6 to 50.8 kg	0.75	−0.29	0.14	−0.68 to 0.10
>50.8 kg	0.47	−0.76	<0.001	−1.14 to −0.37
Source				
Local dairy farms	Referent			
Order buyer	1.61	0.48	0.02	0.07 to 0.88
Auction	0.97	−0.03	0.85	−0.36 to 0.30
Arrival group/room				
0	Referent			
1	0.82	−0.19	0.29	−0.56 to 0.17
2	0.85	−0.16	0.44	−0.58 to 0.26
3	0.88	0.13	0.52	−0.54 to 0.27
Constant	0.09	−2.41	<0.001	−2.97 to −1.84

1 Treatments: CON = all-milk-protein MR; P5 = 15% of CP replaced with spray-dried porcine plasma included at 5% of the absolute formula; P10 = 30% of CP replaced with spray-dried porcine plasma included at 10% of the absolute formula.

Overall, 111 calves (34.7%) were treated (oral meloxicam and trimethoprim sulfadoxine) at least once for diarrhea, with 30.0% of the CON group, 36.2% of the P5 group, and 38.1% in the P10 group being treated. Survival analysis was conducted and showed no statistical differences between treatment groups with respect to diarrhea treatment. Arrival weight and source of calves were associated with calves being treated for diarrhea (Table 2). Of those that received initial treatment, 6.1, 10.5, and 10.0% required further antibiotic treatment due to prolonged diarrhea in the CON, P5, and P10 groups, respectively. The number of calves that required further intervention for diarrhea following the first antibiotic treatment did not differ between groups (*P* = 0.78).

**Table 2:** Cox proportional hazards model evaluating first antibiotic treatment for diarrhea over the 77-d experimental period in 320 calves randomly assigned to receive 1 of 3 milk replacers^1^

Variable	Hazard ratio	*P*-value	95% CI
Treatment group			
CON	Referent		
P5	1.28	0.30	0.80 to 2.05
P10	1.42	0.14	0.89 to 2.26
BW at arrival			
<44.5 kg	Referent		
44.5 to 47.6 kg	0.62	0.07	0.37 to 1.03
47.6 to 50.8 kg	0.72	0.35	0.43 to 1.22
>50.8 kg	0.57	0.04	0.33 to 0.98
Source			
Local dairy farms	Referent		
Order buyer	2.17	0.002	1.34 to 3.53
Auction	1.31	0.25	0.83 to 2.06

1 Treatments: CON = all-milk-protein MR; P5 = 15% of CP replaced with spray-dried porcine plasma included at 5% of the absolute formula; P10 = 30% of CP replaced with spray-dried porcine plasma included at 10% of the absolute formula.

A total of 208 calves (65.0%) were treated once for respiratory disease during the experimental period. In the CON group, 63.6% of the calves were treated, whereas in the P5 and P10 groups, 62.9 and 68.6% of calves were treated, respectively. In a Cox proportional hazards model, no statistical differences among the treatment groups were found with respect to respiratory disease treatment (*P* = 0.40). Arrival weight and source of calves were associated with calves being treated for respiratory disease. Specifically, calves weighing >50.8 kg had a lower hazard (HR: 0.65; 95% CI: 0.43–0.98; *P* = 0.04) of being treated for respiratory disease than calves weighing <44.5 kg at arrival. In addition, calves sourced from auction had a greater hazard (HR: 1.37; 95% CI: 1.00–1.88; *P* = 0.05) than calves sourced from a local dairy farm. Of the calves that were treated once, 64.3, 59.1, and 58.3% in the CON, P5, and P10 groups were treated again for respiratory disease, respectively, with no statistical differences (*P* = 0.74) between groups.

With respect to weekly BW measurements, day of measurement (*P* < 0.001) significantly influenced BW over time, as did BW at arrival and source of calves. Treatment group (*P* = 0.99) and the treatment by day interaction (*P* = 0.41) were not significant influencers of BW over time. However, the BW of calves in the CON (115.8 ± 15.5 kg) group was greater than that of calves in the P5 group (113.4 ± 17.8 kg) at d 77 (*P* = 0.02).

Calf MR intake and refusals and ADG are reported in Table 3. We found no differences between groups with respect to calf MR intake and refusals, ADG preweaning, or ADG over the entire experiment; however, there was a tendency for calves in P5 to have a lower ADG postweaning compared with calves in CON. In the CON, P5, and P10 groups, 106.09 ± 11.54 kg, 104.35 ± 16.09 kg, and 103.54 ± 10.28 kg of grain were consumed by pod over the entire experimental period (*P* = 0.80). Grain intake by pod during the preweaning period was 31.71 ± 7.06 kg, 32.60 ± 8.12 kg, and 31.38 ± 4.67 kg in the CON, P5, and P10 groups, respectively (*P* = 0.97). For the postweaning period, 74.38 ± 6.83 kg, 71.75 ± 10.28 kg, and 72.16 ± 7.62 kg of grain was consumed by pod in the CON, P5, and P10 groups, respectively (*P* = 0.75). Feed efficiency for the entire experimental period was 0.46 ± 0.03, 0.45 ± 0.04, and 0.47 ± 0.05 kg of gain/kg of feed, for the CON, P5, and P10 groups, respectively. There were no differences between treatment groups with respect to feed efficiency over the entire period (CON vs. P5: *P* = 0.40; CON vs. P10: *P* = 0.50). Feed efficiency in the preweaning period (<56 d after arrival) was 0.58 ± 0.04, 0.58 ± 0.05, and 0.59 ± 0.06 kg of gain/kg of feed for the CON, P5, and P10 groups, respectively; no statistical differences were found among groups (CON vs. P5: *P* = 0.77; CON vs. P10: *P* = 0.29). In the postweaning period (56 to 77 d after arrival), 0.36 ± 0.08, 0.32 ± 0.10, and 0.35 ± 0.08 kg of gain/kg of feed was found in the CON, P5 and P10 groups, respectively. No differences were found between the groups (CON vs. P5: *P* = 0.23; CON vs. P10: *P* = 0.97).

**Table 3:** Milk replacer (MR) intake and refusals and ADG preweaning, postweaning, and overall (means ± SD)

Variable	Treatment^1^			*P*-value
	CON	P5	P10	
MR intake, kg	40.89 ± 4.36	40.80 ± 5.36	40.48 ± 6.51	0.50
MR refusals, kg	0.09 ± 0.29	0.08 ± 0.32	0.16 ± 0.62	0.72
ADG, kg/d				
Preweaning (d 1–56)	0.75 ± 0.17	0.76 ± 0.21	0.75 ± 0.17	0.84
Postweaning (d 56–77)	1.19 ± 0.51	1.04 ± 0.51	1.15 ± 0.47	0.08
Total (d 1–77)	0.87 ± 0.18	0.83 ± 0.22	0.87 ± 0.17	0.42

1 Treatments: CON = all-milk-protein MR; P5 = 15% of CP replaced with spray-dried porcine plasma included at 5% of the absolute formula; P10 = 30% of CP replaced with spray-dried porcine plasma included at 10% of the absolute formula.

In this experiment, we observed few differences between treatment groups. There were no differences found in treatment of disease, feed conversion, or mortality. Slight differences were observed in fecal consistency score, with P10 having a tendency for a higher proportion of days with a fecal score of ≥2 compared with the CON group. These results are in contrast to disease-challenged, milk-fed calf studies demonstrating that the supplementation of spray-dried plasma-based proteins improve gut health (Nollet et al., 1999; Hunt et al., 2002). Specifically, supplementing spray-dried bovine serum at 57 g/d reduced diarrhea volume and oocyst shedding. In addition, when using a chromium EDTA model combined with measures of villous surface area and crypt depth after euthanizing calves at 18 d of age, gut integrity was improved in calves supplemented with plasma-based proteins and orally challenged with *Cryptosporidium parvum* (Hunt et al., 2002). Furthermore, research examining supplementation of either spray-dried bovine or porcine plasma fed at 75 g/d demonstrated reduced calf mortality and pathogen shedding when colostrum-deprived calves were challenged with virulent strains of *Escherichia coli* (Nollet et al., 1999). In this study, we hypothesize that there was no effect on enteric disease from supplementing spray-dried plasma because of the relatively minimal incidence of severe diarrhea (fecal score of 3 occurred in only 1.6 to 2.2% of the calves, depending on the treatment group) and relatively low overall calf mortality (3.4%), with just 4 calves in the entire study dying from diarrhea. Enteric disease challenge was greater in these other studies. Significant respiratory disease challenge did occur in this trial (65% of calves were treated for respiratory disease) but feeding spray-dried porcine plasma (Wood et al., 2019) or spray-dried bovine plasma (Wood et al., 2013) to calves has not been shown to affect its incidence.

When evaluating growth, no differences were found between treatment groups over most of the BW measurements; however, a difference between P5 and CON was found on d 77. Improvement in ADG had been noted previously when feeding spray-dried bovine plasma at 3.3% of the absolute MR formula in calves orally challenged with virulent strains of *E. coli* (Quigley and Drew, 2000). This may indicate that improvements in ADG from plasma supplementation only occur when the calf is under duress of severe gut health maladies, which was not the case in this study.

Market values for milk-based and plasma-based proteins are variable, and sometimes the replacement of milk proteins with SDPP is economically advantageous. Also, the incidence and severity of enteric-disease challenge is variable and unpredictable, particularly in commingled calves sourced from auctions and order buyers. Therefore, because inclusion of SDPP in MR has previously been shown to reduce mortality and morbidity in calves under duress of more severe enteric-disease challenge than was experienced in this research trial, it warrants consideration for inclusion in calf milk replacers.
